# A novel algorithm- specific loci screening accelerates the establishment of molecular quantification of *Glycyrrhiza glabra*, *G. uralensis*, and *G. inflata*

**DOI:** 10.1186/s13020-025-01263-2

**Published:** 2025-11-24

**Authors:** Yifei Pei, Ziyi Liu, Wenjun Jiang, Mingyu Zhang, Haitao Liu, Xue Feng, Xiwen Li

**Affiliations:** 1 State Key Laboratory for Quality Ensurance and Sustainable Use of Dao-Di Herbs, Institute of Chinese Materia Medica, China Academy of Chinese Medical Sciences, Beijing, 100700 China; 2 Institute of Medicinal Plant Development, Chinese Academy of Medical Sciences, Peking Union Medical College, Beijing, 100193 China

**Keywords:** Species quantification, Pyrosequencing, Chloroplast genome, Single nucleotide polymorphism; *Glycyrrhiza*

## Abstract

**Objective:**

This study aims to establish a specialized molecular method for distinguishing different licorice and quantifying target species accurately from mixtures.

**Methods:**

A rapid specific loci screening (SLS) algorithm was developed in this study, and used the chloroplast genome sequences were used to screen for variable loci in the *Glycyrrhiza glabra* (GG), *G. uralensis* (GU), and *G. inflata* (GI). Each locus has been validated by polymerase chain reaction and pyrosequencing. The selected loci were analyzed by Herb molecular quantification (Herb-Q) assay to complete the quantitative methodology verification and establish a quantitative detection system for homemade licorice mixtures and one of the patent medicines—Liuyi San.

**Results:**

Outstanding performance was observed in quantitative validation, which evaluated linearity (0.9989), limit of detection (2%) and quantification (2%), and repeatability; additionally, a quantitative detection system was established for homemade licorice mixtures and Liuyi San (one of the patent medicines), with the lowest bias being 3.48%. When both GG and GU were present in the mixed powder, the average biases for GG and GU quantification were 8.25% and 8.01%, respectively. When only GG was present in Liuyi San, the bias was 6.43%; when both GG and GU were present, the biases for GG and GU were 5.61% and 3.48%, respectively.

**Conclusion:**

This study successfully established an accurate detection system for quantifying the botanical origin of edible-medicinal licorice, which represents a significant milestone in enhancing the safety, efficacy, and quality control in licorice products.

**Supplementary Information:**

The online version contains supplementary material available at 10.1186/s13020-025-01263-2.

## Introduction

Herbs have played a significant role in countries where traditional medicine dominates, and they also serve as dietary supplements in Western medicine-oriented nations [1, 10, 37]. Licorice, a perennial herb used for both medicinal and edible purposes, belongs to the genus *Glycyrrhiza* in the family Fabaceae [6]. In the pharmacopoeias of China, the United Kingdom, South Korea, and Europe, *Glycyrrhiza uralensis* Fisch (GU), *G. glabra* Linn (GG), and *G. inflata* Batalin (GI) are recorded as the three origin species of medicinal licorice [35]. The first two species are included in the pharmacopoeias of the United States and Japan, while only GU is permitted in the Indian pharmacopoeia [35]. Mixing different medicinal licorice species with different chemical compositions will damage the consistency and stability of their efficacy, which is not conducive to clinical precision medicine. For example, through comparative analysis of genetic and chemical markers of three medicinal licorice species, glabridin was confirmed as a species-specific secondary metabolite of GG [30]. Fold differences in the effects on the metabolism and clearance of CYP3A4 and CYP1A2 substrates drugs are observed among different licorice species [15].

In clinical applications, controlling the use and dosage of individual licorice species in different prescriptions or batches of medicinal materials is particularly crucial for ensuring the efficacy consistency and quality control of licorice medicinal materials [7]. Currently, research on licorice medicinal species remains primarily focused on the identification of chemical components [17, 35]. By contrast, reports on adulteration detection are relatively scarce, and such detection methods also face challenges in practical application and popularization. High-resolution melting analysis (HRM) can examine the genotype of simple sequence repeat sites and provide a set of reliable and robust simple sequence repeat markers (SSRs) for species identification of GU, GG, GI, and *G. spinulosa*, but it has failed to obtain species-level quantitative detection results [20]. Near-infrared spectroscopy is used to construct a classification model to achieve rapid and non-destructive identification of GU and *G. pallidiflora* [11]. However, this method faces challenges in practical application and promotion due to limitations of the input dataset. Currently, research on species-level identification and quantification of licorice remains relatively limited, with no reports available on species-level quantitative detection of medicinal licorice in traditional Chinese patent medicines.

Herb-Q, an effective tool for low-taxonomic-level analyses that relies on species-specific loci, is well-suited for qualitative and quantitative detection for herbs [12, 18, 24]. Nevertheless, it faces a critical bottleneck: the manual screening of suitable loci is tedious, error-prone, and time-consuming [18, 24, 25]. Although the chloroplast (cp) genome—including regions such as *ITS*2, *mat*K, *psb*A-*trn*H, and *rbc*L [5, 13, 14]—provides candidate gene loci for medicinal licorice [19], the large volume of data generated via high-throughput sequencing further increases screening costs in the absence of automated tools. Accordingly, there is an urgent need to create a computer-based procedure that can quickly screen and confirm species-specific locus tailored for Herb-Q assay.

Herein, we developed a new algorithm for specific site screening (SLS) and verified the feasibility of the loci recommended by the process through qualitative and quantitative experiments. At the same time, Herb-Q identification and quantification detection system suitable for edible-medicinal licorice was established, and its quantification ability of the system in mixtures and patent medicines was verified.

## Materials and methods

### Sample collection and authentic verification

A total of 45 root samples of three medicinal licorice species *G. uralensis* (GU), *G. glabra* (GG). and *G. inflata* (GI) were collected from Gansu, Henan, Shaanxi, Xinjiang Provinces and Nei Mongol, Ningxia Hui Autonomous regions in China, respectively (Table S1: sample information). All these samples were identified as by Professor Xiwen Li (Institute of Chinese Materia Medica, China Academy of Chinese Medical Sciences). Separately clean, slice, and dry each licorice sample to a constant weight, then grind into a fine powder and sift through a 100-mesh sieve for use. Their authenticity was ensured by Sanger sequencing and BLAST alignment of the ITS2 region and *ndhC*-*trnV* region. High quality DNA stock solution was extracted by the Universal Genomic DNA Kit (Tiangen Biotech, Beijing Co., China). The reaction system and PCR procedure followed the primers ITS2-S2F/S3R method and *ndhC*-*trnV* method [3] and were performed on an Applied Biosystems 2720 Thermal Cycler (Applied Biosystem, Madrid, Spain). The PCR products performed Sanger sequencing at Tsingke Biotechnology Co., Ltd. (China) and then the sequences were aligned in BLAST (https://blast.ncbi.nlm.nih.gov/Blast.cgi).

### Phylogenetic analysis

The chloroplast genome sequences were downloaded from NCBI database. All sequences were used MAFFT (version 7.453) to obtain the alignment sequence, which was used to establish the maximum likelihood tree by RAxML (version 8.2.12) with 1000 ultrafast bootstrap replicates using the GTRGAMMA model.

### SLS algorithm to select specific locus for Herb-Q assay

**Fig. 1:**
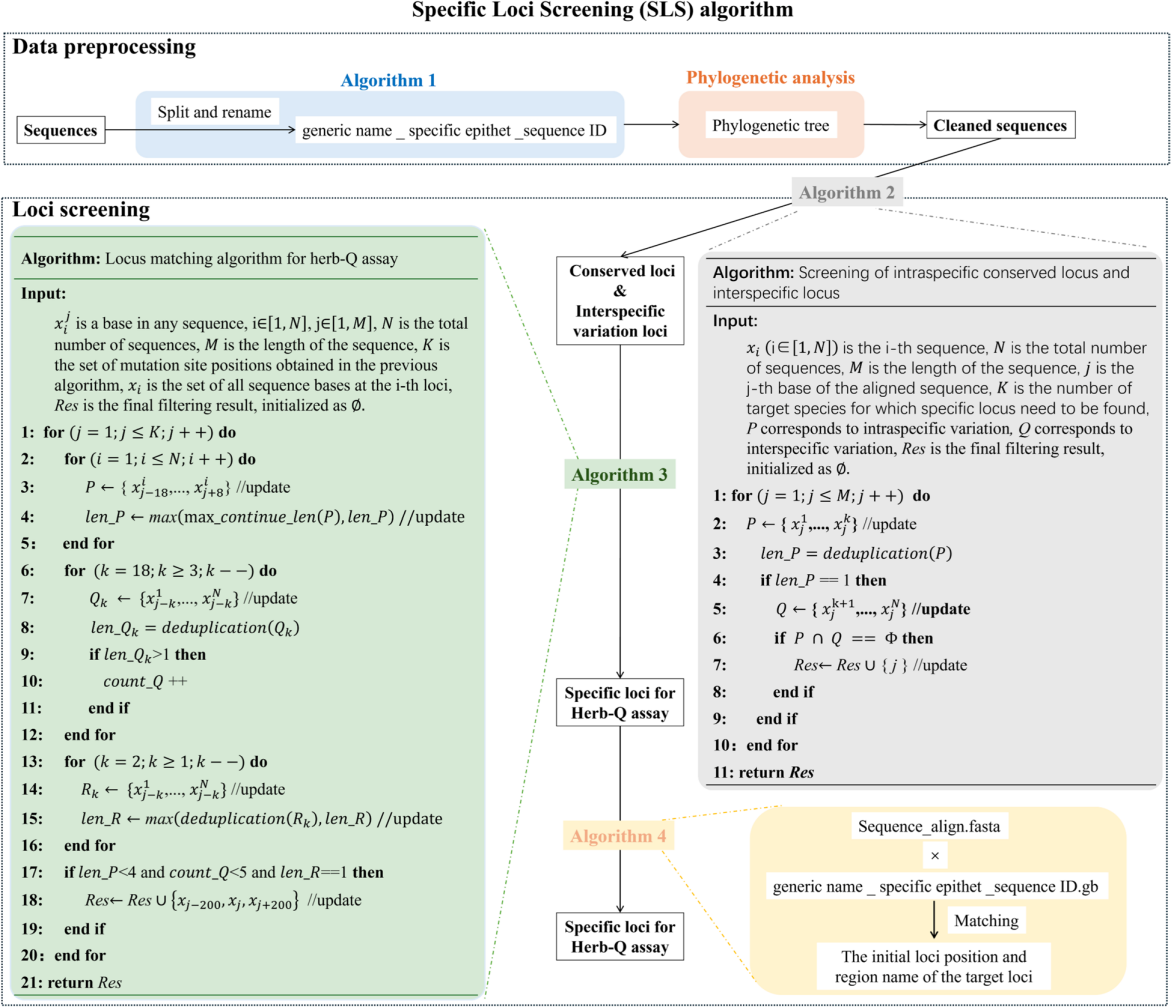
Flow chart of the Specific Loci Screening (SLS) algorithm and the algorithm of algorithm 1 to algorithm 4. The SLS algorithm consists of two parts: data preprocessing and site screening

A new SLS algorithm was established to help quickly and accurately screen the Herb-Q-specific sites suitable for medicinal licorice in the plastid genome. The SLS algorithm first completes data cleaning and label unification of the input data through data preprocessing, and then screens specific sites based on the requirements for identification sites according to the Herb-Q assay. The overall idea of the SLS algorithm is shown in Fig. 1, which includes the key content of data preprocessing and the content of the four main self-built algorithms used for loci screening.

Data preprocessing: Given the well-established evolutionary relationships among licorice (Glycyrrhiza spp.) species, the construction of a phylogenetic tree to clarify the evolutionary position of each cp sequence was adopted as a core step in data preprocessing. This preprocessing step ensured the accuracy and reliability of the input cp sequence dataset for subsequent site screening analyses.

**Fig. 2:**
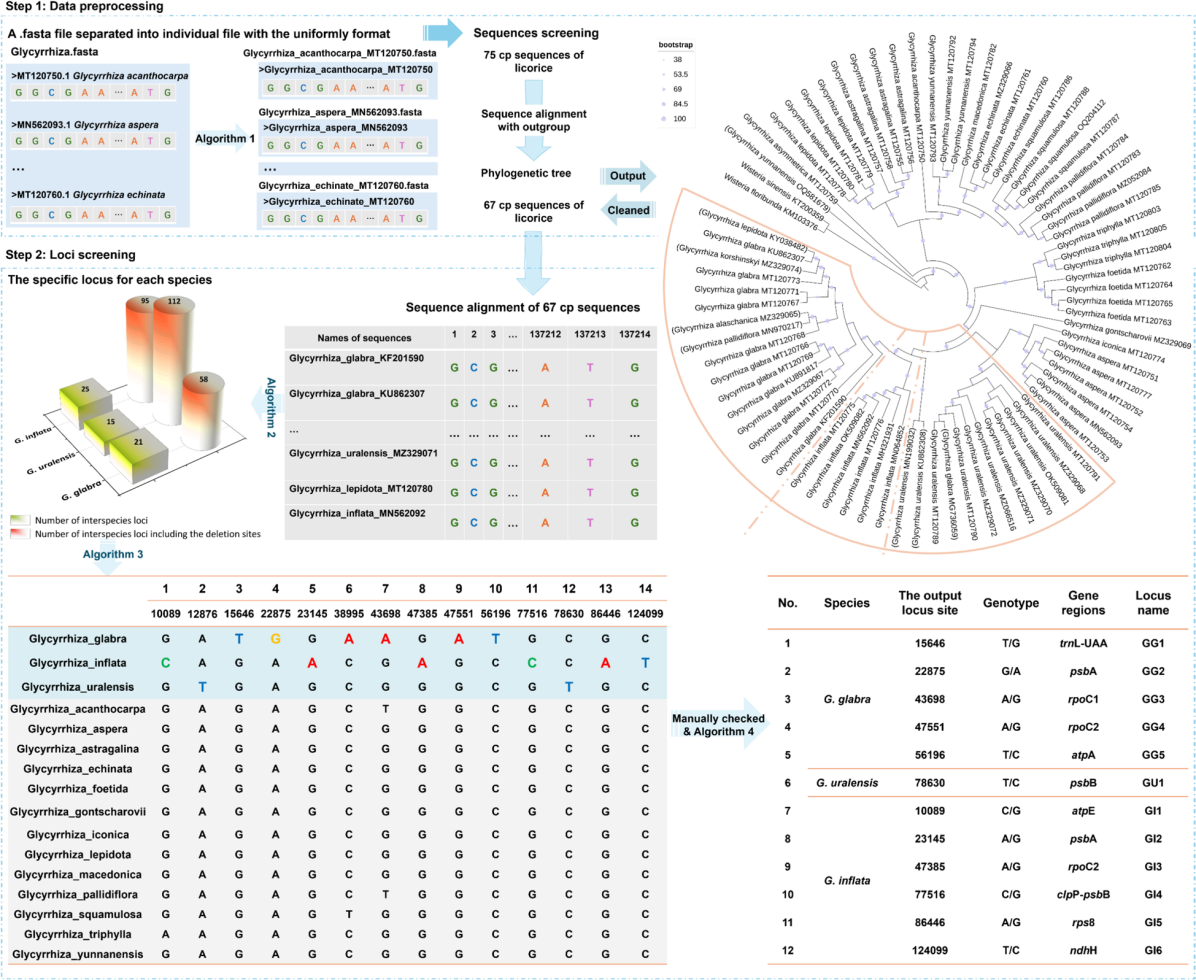
The results of loci screening of GG, GU and GI based on SLS algorithm. 75 chloroplast genome sequence data of genus *Glycyrrhiza* were preprocessed by Algorithm 1 and phylogenetic analysis, and 67 sequences were obtained to form a two-dimensional data matrix of [67 × 137214]. Algorithm 2 was used to screen loci that were specific to all species in the genus *Glycyrrhiza*. Algorithm 3 was developed based on the basic requirements of pyrosequencing and loci suitable for the Herb-Q assay were screened for the GG, GU and GI. On this basis, 12 loci were finally obtained by manually checking special cases such as AT-rich regions, and the molecular feature information of these loci was output by Algorithm 4

Loci screening: Four main self-built algorithms completed the loci screening work for qualitative and quantitative detection of Herb-Q assay. Algorithm 1 was used to split all sequences into single sequence files, and all sequences are renamed to the format of “generic name _ specific epithet _sequence ID”. Algorithm 2 was created to obtain information on conserved sites within a single species and interspecific variation sites. Algorithm 3 was developed to screen specific sites suitable for the pyrosequencing-based Herb-Q assay. These loci were screened out through a three-step judgment criterion (Fig. 2): (1) In the sequence region interval [locus-18, locus + 8], there are no sites with four or more consecutive identical bases; (2) In the sequence region interval [locus-2, locus-1], there are no variant sites among all sequences; (3) In the sequence region interval [locus-18, locus-3], there are no variant sites among all sequences more than five. The function of algorithm 4 is to extract the sequence feature information of specific sites in the original sequence data. Furthermore, the specific codes of the four self-developed algorithms and the files describing their computational steps, parameters, and validation process can be found in the Supplementary code files.

### Method validation

One of the specific sites obtained by our algorithm for GG was selected for quantitative methodology validation, while GG corresponded to the target species samples, and a 1:1 mixture of the other two species was considered as the adulteration samples. The range of quantitation was determined using adulterated samples at 1, 2, 4, 6, 8, 10, 20, 30, 40, and 50% (w/w) and the corresponding proportions of the target species samples were 99, 98, 96, 94, 92, 90, 80, 70, 60, and 50% (wt/wt). The limit of detection (LOD) and limit of quantification (LOQ) were evaluated using adulterated samples at 8, 6, 4, 2, and 1% and the corresponding proportions of the target species samples were 92, 94, 96, 98, and 99%. The accuracy and repeatability were evaluated using adulterated samples at 15, 25, and 35% and the corresponding proportions of the target species samples were 85, 75, and 65%. All mixtures were purified using a Universal Genomic DNA Kit to obtain high quality DNA stock solution, and pyrosequencing was performed after obtaining PCR products. The optimized suitable reaction system and PCR procedure are detailed in Table S2: PCR and sequencing primers for pyrosequencing. The pyrosequencing procedure and methods of developmental validation as same as our previous research [24].

### Sample preparation for Herb-Q assay application

A licorice species can be randomly selected as an external standard for the species-level quantification for medicinal licorice. Here, the addition of a known weight of GI as an external standard was used as an example for experimental verification. We used self-prepared mixtures and Liuyi San as research materials to quantification experiments. Dextrin or mineral substances used as adjuvants were randomly added to samples M1 to M3 to simulate the contamination or preliminary treatment of the samples. Samples L1 to L3 are composed only of talc and (or) medicinal licorice. The specific proportions of each sample were as follows. Sample M1 was composed of 66.7 mg GG powder, 33.5 mg GU powder, 20.1 mg mineral substances, and 150.3 mg GI powder, with a total weight of 270.6 mg. Sample M2 consisted of 66.6 mg GG powder, 33.4 mg GU powder, 20.1 mg dextrin, and 150.0 mg GI powder, with a total weight of 270.1 mg. Sample M3 was made up of 66.5 mg GG powder, 33.9 mg GU powder, 20.0 mg soil and starch, and 150.4 mg GI powder, with a total weight of 270.8 mg. Sample L1 was 200.1 mg of pure talc. The sample L2 was a mixture of 40.2 mg GG powder, 240.1 mg talc, and 60.1 mg GI powder, with a total weight of 340.4 mg. Sample L3 consisted of 12.1 mg GG powder, 36.1 mg GU powder, 288.0 mg talc, and 52.1 mg GI powder, with a total weight of 388.3 mg. The calculated methods are the same as the previous studies. The total weights of GG, GU, and GI were calculated first as in (1), and the weight of GG or GU (W_target) were obtained as in Eq. (2) below. The bias should be less than 25% and could be calculated as in Eq. (3).

1 $$W\_licorice = W\_GI / R\_GI$$

2 $$W\_target = W\_licorice \times R\_target$$

3 $$bias = \frac{{\left| {W\_target - A\_target} \right|}}{A\_target} \times 100\%$$

where W_GI represents the actual weight of GI in each sample; R_GI is the ratio of chemiluminescent signals of unique genotype of GI at one locus; W_licorice is the calculated total amounts of GG, GU, and GI; and R_target is the ratio of the chemiluminescent signal of a unique genotype for GG or GU at two loci respectively; A_target represents the actual weight of target species in the test sample and W_target is the calculated weight of target species in the test sample.

## Results

### Selection of specific locus in licorice for Herb-Q assay by SLS algorithm

#### Sequence data preprocessing

The 75 cp sequences of genus *Glycyrrhiza* downloaded from NCBI (Table S3: Collection of chloroplast genome sequences of genus *Glycyrrhiza*) were used to establish a phylogenetic tree to verify the cp sequences for the next step of candidate specific locus selection, together with *Wisteria sinensis*, *W. floribunda* as an outgroup. First, all sequences were split into single sequence files using algorithm 1 (Fig. 2), and all sequences were renamed in the format of “generic name _ specific epithet _ sequence ID”. In addition, the names of species with updated Latin names in the above sequences were uniformly modified to the latest naming method (Table S3) before subsequent analysis. The result of the phylogenetic tree shows that eight sequences may be mistakenly uploaded to the public database (Fig. 2), including *G. yunnanensis* (OQ581697), *G. lepidota* (KY038482), *G. pallidiflora* (MN970217), *G. uralensis* (MZ329074, MZ329065, MN199032 and KU862308) and *G. glabra* (MG736059). Further, the remaining sequences were re-aligned to a matrix of 137,214 bp (columns) × 67 sequences (rows), which were used as the input data file for the next step.

#### Specific locus recommended for qualitative use with the Herb-Q assay

The developed algorithm 2 (Fig. 2) successfully screened out specific loci with intraspecies conservation and interspecies variation characteristics for the three medicinal licorice species. A total of 95, 112 and 58 sites were found as the specific sites for GI, GU and GG, respectively (Fig. 2, Tables S4-S6: The total loci result of GI, GU and GG output from algorithm 2). However, the deletion loci “-“ are not conducive to subsequent qualitative identification, especially further quantitative detection. Therefore, priority was given to selecting specific sites with only base variations. Algorithm 2 screened out 25, 15, and 21 sites for GI, GU, and GG, respectively (Fig. 2, Tables S7-S9: The loci result with only base variations of GI, GU and GG output from algorithm 2). Further, the above specific locus for each species will be used as input data for algorithm 3, and sites suitable for the Herb-Q method will be screened out through a three-step judgment criterion (Fig. 2). In addition, the unique sites in the AT-rich region were not applicable. Finally, the numbers of specific sites suitable for qualitative analysis via the Herb-Q assay, obtained from the final screening, were 6, 1 and 5 for GI, GU and GG, respectively (Fig. 2, Tables S10-S12: Loci results of GI, GU and GG output from algorithm 3).

To facilitate the subsequent primer design, the sequence information of each locus from 200 bp to the last 200 bp in the original cp sequence was also output. Algorithm 4 also outputs information on the gene position of each site in the original cp sequence (Fig. 2, Tables S13-S15: Loci results of GI, GU and GG output from algorithm 4). Among the 12 loci output for Herb-Q assay, half were G/A mutations, a quarter were T/C mutations, G/C and T/G mutations had also occurred. In addition, all above loci were located on the gene coding region.

#### Specific locus recommended for quantitative use with the Herb-Q assay

The specific loci for each species obtained theoretically through the SLS algorithm cannot be fully confirmed to be truly usable, so it is necessary to further verify the 12 obtained loci by experimentally testing (Figure S1: Verification of the specific single nucleotide polymorphism loci in GG, GU, GI and the mixture at the 12 loci). For the 10,089 locus (named GI1), three possible detection outcomes were anticipated. The first outcome is the display of only the chemiluminescence signal peak corresponding to base C, followed by the second outcome, which is the display of only the peak associated with base G. The third outcome is the concurrent display of both peaks, representing base C and G. Our experimental results demonstrate that when the test sample is GI, it exhibits the first expected result. In contrast, when the test sample is either GG or GU, it displays the second expected result. Notably, when the test sample consists of a mixture of GG, GU, and GI, the results align with the third expected outcome. The other 11 loci also obtained 3 types of expected results, and the results of all tested samples were consistent with their respective expected results. At the same time, the baselines of all pyrograms of all loci showed acceptable smoothness. Therefore, the applicability of the 12 loci obtained by the SLS algorithm in the qualitative analysis of Herb-Q assay is 100%. Based on the preference for specific loci that do not contain the base A [16], among the 12 loci mentioned, 2 loci (GG1 and GG5), 1 locus (GU1), and 3 loci (GI1, GI4 and GI6) were considered more suitable as quantitative detection loci for the Herb-Q assay for GG, GU and GI, respectively.

### Methodological testing of quantitation for medicinal licorice

**Fig. 3:**
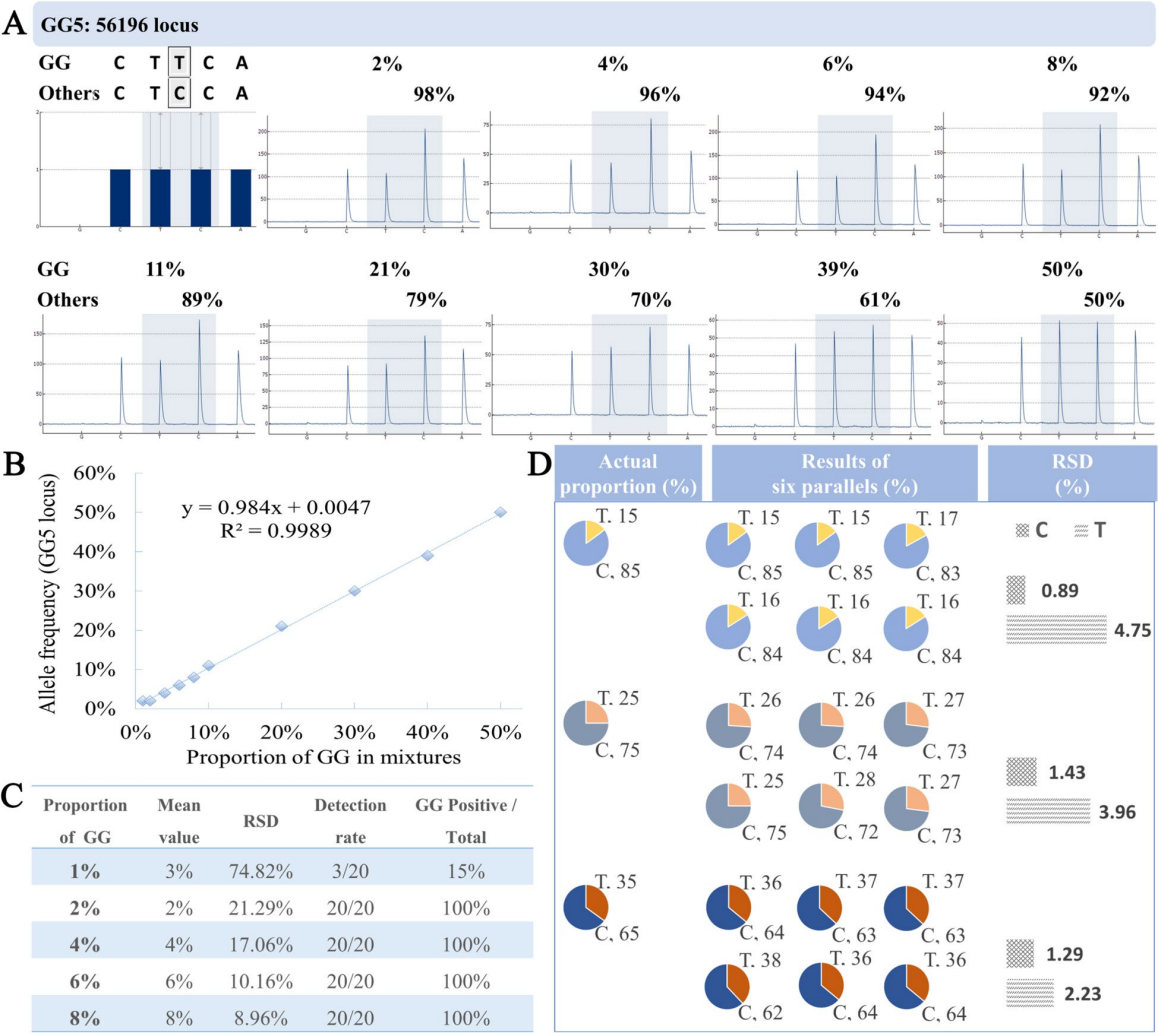
Pyrograms of 10 different proportions at the GG5 (56,196) locus and the qualitative results for Herb-Q. **A**1 are the expected pyrogram and the pyrograms of mixtures at the GG5 locus with GG powder at proportions of 50, 40, 30, 20, 10, 8, 6, 4, and 2% (wt/wt) in the mixture samples, respectively. **B** is the regression equation of GG in mixture samples, with R^2^ being 0.9989. **C** shows the limit of detection and limit of quantitation for GG in mixture samples by Herb-Q (n = 20). **D** is the verification results of quantification for samples with known proportions. GG, *G. glabra*. GI, *G. inflata*. The mixture samples are the mixtures of GG and GI powder

The validation of the Herb-Q methodology was completed using the specific site 56,196 locus (GG5) of GG as an example, including linearity, limit of detection (LOD), limit of quantification (LOQ) and repeatability. Ten different proportioned mixtures of GG and GI powder samples were evaluated for their linear relationship between the expected genotype frequency and the measured fluorescence signal values through pyrosequencing results (Fig. 3A, Table S16: The raw data of all proportions for Linearity) using the Herb-Q assay. All proportions yielded accurate detection of the corresponding genotype, except for the 1% GG mixture samples. The coefficient of determination was 0.9989, indicating a standard linear relationship between the expected genotype frequencies and the measured values (Fig. 3B). The LOD and LOQ of this study were determined by the detection rate results and RSD values of the lowest proportion of 20 experiments (Table S17: The raw data of all proportions for LOD/LOQ), respectively. The detection rate of the mixture samples of 1% GG and 99% GI was less than 95%, and the RSD of the mixture samples of 2% GG and 98% GI was less than 25% (Fig. 3C). Therefore, the LOD and LOQ of the mixtures of the Herb-Q assay for the quantitative detection of medicinal licorice at the species level were both 2%. The mixtures of GG (15, 25 and 35%) and GI (85, 75 and 65%) were used as samples, and the repeatability of this method was judged by the RSD values of the results of 6 replicates. The average results of GG in the three proportion samples were 16, 27 and 37%, and the calculated RSDs were 4.75, 3.96 and 2.23%, respectively (Fig. 3D). The average results of GI were 84, 73 and 63%, and the calculated RSDs were 0.89, 1.43 and 1.29%, respectively (Fig. 3D). The RSD values of all the results were less than 25%, indicating that the method had good reproducibility.

### Calculation of quantification results for self-prepared samples

**Fig. 4:**
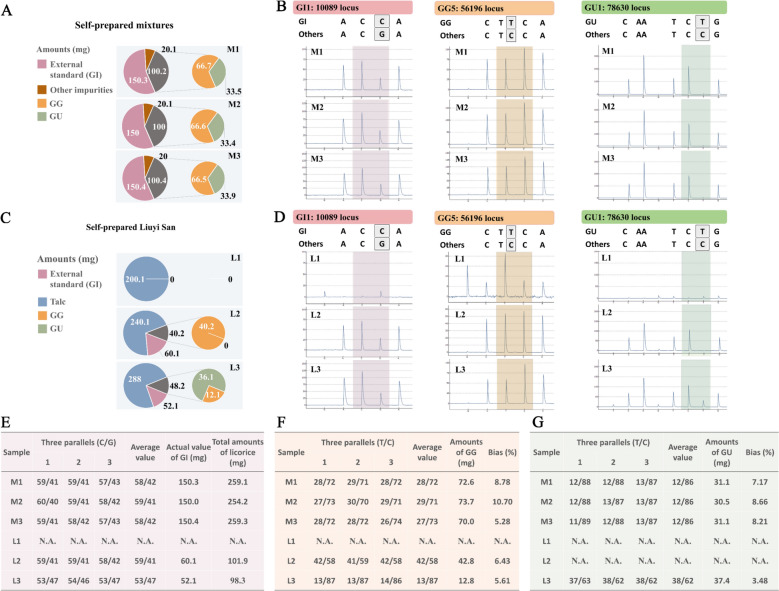
Flowchart and results of the Herb-Q approach for detecting self-prepared licorice mixtures and Liuyi San. The true values of components making up samples M1-M3 (**A**) and L1-L3 (**C**) based on weight. The pyrograms of M1-M3 (**B**) and L1-L3 (**D**) at the GI1 (10,089) locus, GG5 (56,196) locus and GU1 (78,630) locus. The values of three parallels, average values, actual values of GI and total amounts of licorice at the GI1 locus of samples M1-M3 and L1-L3 (**E**). The values of three parallels, average values, actual values of GG and total amounts of licorice at the GG5 locus of 6 samples (**F**). The values of three parallels, average values, actual values of GU and total amounts of licorice at the GU1 locus of test samples (**G**). GG, *G. glabra*. GU, *G. uralensis*. GI, *G. inflata*. M1-M3 are the self-prepared mixtures and L1-L3 are the self-prepared Liuyi San

Three mixture samples (M1 to M3) and three Liuyi San samples (L1 to L3) were prepared to evaluate the quantitative detection effect of the specific loci selected by SLS. The component contents of each sample are shown in Fig. 4A, C. Pyrograms of the pyrophosphate sequencing results for these samples are displayed in Fig. 4B, D.

The W_licorice of M1 to M3 samples were calculated using the chemiluminescent signal ratio of base C/G at the GI1 locus (Fig. 4E) and formula 1, yielding values of 259.1, 254.2, and 259.3 mg, respectively. W_GG and W_GU can be calculated using the chemiluminescent signal ratio of genotype T at the GG5 and GU1 loci, respectively. M1 to M3 samples showed average R_GG values of 72, 71 and 73% at the GG5 locus (Fig. 4F), and average R_GU values of both 12% at the GU1 locus (Fig. 4G). The average measured values of W_GG of samples M1 to M3 estimated by formula 2 (Fig. 4F) were 72.6, 73.7, and 70.0 mg, respectively, while the actual measured values were 66.7, 66.6, and 66.5 mg, respectively; the average measured values of W_GU (Fig. 4G) were 31.3, 30.5, and 31.1 mg, respectively, while the actual measured values were 33.5, 33.4, and 33.9 mg, respectively. The bias values of samples M1 to M3 at the GG5 locus calculated by formula 3 were 8.78, 10.70, and 5.28%, respectively, all below 25%. The bias values of samples M1 to M3 at the GU1 locus calculated by formula 3 were all below 25%, which were 7.17, 8.66, and 8.21%, respectively.

The determination and calculation methods of L1 to L3 samples were the same as those of M1 to M3 samples. L1 sample was a negative control, so no results consistent with the expected base were detected at the three loci. The talc powder of L2 sample was only made into Liuyi San with GG, so the determination values of W_licorice and W_GG can be calculated based on the results of GI1 and GG5 loci respectively (Fig. 4E, F), and the bias value of the target species GG in L2 sample was 6.43%. The bias values of GG and GU in Liuyi San L3 samples based on the results of the three loci, and the bias values were 5.61% and 3.48% respectively.

## Discussion

### Comparison of molecular versus chemical methods and the core role of locus selection in Herb-Q assay

Both molecular and chemical methods were considered as the common and widely used identification approach for species adulteration analysis, while the former has been used to certify various herb species in the pharmaceutical industry and the latter is more often included in the pharmacopeias of various countries [35]. Although chemical methods hold a significant advantage in identifying and quantifying indicator chemical components within herbs, due to the particularity of the efficacy of herb medicines, the detection of single or multiple characteristic components cannot fully represent the herb materials themselves [32]. When two medicinal materials with similar chemical compositions are mixed [22] or characteristic component extracts from other plants are added to the extracts of the target medicinal materials[23], chemical methods for herb quantification tend to produce false results, limiting their species-level identification and quantitative analysis. In contrast, molecular methods [36, 33], especially the Herb-Q assay [18, 29], show significant advantages in species identification, a point increasingly recognized by scholars who consider species quantification detection a crucial yet underappreciated aspect of quality control in traditional Chinese medicine product development.

Herb-Q has been proven to be effective in quantifying closely related species in* Pinellia ternata* [28], and is expected to become a user-friendly and accurate method for quantifying herb products, food products, agricultural products and environmental samples. Identifying the suitable species-specific locus is a crucial first step in quantifying herbs using Herb-Q assay, while inappropriate locus can result in erroneous output data and even a large bias value [16, 24, 29]. For herbs, the ITS2 sequence region, the *mat*K, *psb*A-*trn*H and *rbc*L regions of chloroplast (cp) genome sequences are commonly considered to be relatively conserved and can be used as DNA barcodes [4, 26, 27]. Many locus-based herb identification or quantification methods also prioritize suitable sites in these regions [5, 13, 14]. The plastid genome provides more opportunities for the selection of suitable loci for the Herb-Q assay, but it also increases the time cost and operation cost of screening. With high-throughput sequencing advancements, multiple cp genome versions have been obtained for the three licorice varieties [19], indicating cp sequences are more suitable as candidate sequences for species-level licorice identification and quantification via Herb-Q assay. However, manual screening of loci meeting quantitative sequencing requirements is cumbersome, labor-intensive, and error-prone, creating an urgent need for a computer-based procedure to rapidly screen and confirm species-specific loci tailored to the Herb-Q assay—addressing this gap is where our SLS algorithm contributes.

### Design, advantages, and limitations of the SLS algorithm

This study used medicinal licorice as the experimental material and developed an SLS algorithm with high compatibility for data processing and screening mainly for the Herb-Q assay. And successfully identified 12 specific loci for Herb-Q quantitative detection for three genus species of medicinal licorice. The SLS algorithm comprises data preprocessing and four major self-developed locus screening algorithms. Data preprocessing is based on two key considerations: (a) sequences stored in public databases often lack verification from data management centers, leading to potential inaccuracies, and verifying the evolutionary position of each cp sequence can effectively filter out or correct such erroneous data; (b) previous phylogenetic analyses of *Glycyrrhiza* plants [8, 35] have provided a reliable reference framework—by aligning newly obtained cp sequences with this framework, sequences with ambiguous or inconsistent evolutionary positions can be identified, excluded, or modified, ensuring the accuracy and reliability of the input cp sequence dataset for subsequent locus screening analyses.

The loci suitable for qualitative and quantitative analysis using the Herb-Q method were primarily screened through four self-developed algorithms: Algorithm 1 splits all sequences and renames them in a unified format, which standardizes sequence naming and structure to facilitate seamless data analysis in subsequent algorithms, eliminate inconsistencies caused by non-uniform sequence formats, and ensure the smooth progression of the entire screening workflow. Algorithm 2 focuses on screening variant locus information from aligned sequences and retains only loci with single nucleotide polymorphism (SNP) variations, a design specifically tailored to pyrosequencing -given the technology’s high sensitivity and specificity for SNP detection and low compatibility with other variation types (e.g., insertions/deletions)—thus ensuring the screened loci are technically feasible for subsequent pyrosequencing-based analyses. Algorithm 3 is dedicated to screening specific loci suitable for pyrosequencing-based Herb-Q detection via a three-step criterion, with all optimizations targeted based on technical principles [16, 18, 24, 29]: the first criterion (“Within the sequence interval [locus-18, locus + 8], there are no sites containing four or more consecutive identical bases”) addresses the inherent limitation of pyrosequencing in homopolymer detection (where signal overlap lead to misjudgment of base count). Here, [locus-18] reserves space for primer design to ensure specific primer-template binding, while [locus + 8] limits the target fragment range to enhance sequencing accuracy and consistency. The second criterion (“Within the sequence interval [locus-2, locus-1], there are no variant sites across all sequences”) guarantees accurate sequencing initiation by preventing primer binding misalignment or shifts in base incorporation caused by adjacent locus polymorphism—critical for pyrosequencing, which relies on precise initial base incorporation. The third criterion (“Within the sequence interval [locus-18, locus-3], the number of variant sites across all sequences does not exceed five”) enhances primer applicability to closely related species and reduces non-specific binding. By controlling variations in the primer-binding region and its adjacent segments, this criterion improves the accuracy of cross-species detection. Algorithm 4 extracts sequence feature information of specific loci from raw sequence data to systematically integrate key biological characteristics (e.g., base composition, adjacent sequence conservation, variation patterns), providing a standardized feature dataset for subsequent Herb-Q detection verification, locus-specificity evaluation, and cross-sample comparison while laying a foundation for algorithm model optimization/iteration and result interpretability analysis.

Compared to existing methods like HRM, which fails to achieve species-level quantitative detection [20], and near-infrared spectroscopy, which faces practical application and promotion challenges due to input dataset limitations [11], the SLS algorithm-enabled Herb-Q assay offers faster screening speed (automated vs. manual locus screening) and higher accuracy (100% species identification accuracy, LOD/LOQ of 2%, and low bias in mixed powders and patent medicines), while reducing labor costs associated with manual screening [24]. However, the loci screened for licorice via SLS algorithm have limitations. First, while this study provided quantitative loci for the three medicinal licorice species using 75 complete chloroplast genomes (i.e., genomic information from 75 individual plants within the *Glycyrrhiza* genus), this sample size is relatively limited compared to the number of samples in real-world scenarios; the currently screened cp genome-specific loci are suitable for medicinal licorice quantitative detection, but may require updates as species evolve and diversity changes to maintain practicality for market. Second, the study focuses on medicinal licorice, while market licorice adulteration involves non-pharmacopeial species (e.g., *G. aspera* Pall., *G. pallidiflora* Maxim., and *G. echinata* L.) [8, 11, 21, 35] that may be misused, large-scale screening of quantitative loci for these adulterants has not yet been completed due to the current unavailability of sufficient genomic sequences, and the adulteration detection system for such cases needs further exploration.

Notably, the selection of GI as the external standard in this study was a result of matching “test sample composition” with “external standard criteria,” not a method-specific constraint. The method is not limited to a single external standard species—eg, GU, GG, or GI can all be used, provided they meet the two core criteria. Criterion 1: The species must have unique SNPs (screened via our SLS algorithm) that enable clear qualitative and quantification via pyrosequencing. Criterion 2: Being absent from the test sample/mixture. The only requirement is to adjust the external standard selection based on the actual composition of the test sample, confirmed via preliminary qualitative analysis.

### Interdisciplinary applications and future research directions

Herb-Q detection technology, combining pyrosequencing with SLS algorithms, boasts strong interdisciplinary applicability and is expected to push the boundaries of traditional herbal medicine quality control, providing targeted solutions across multiple fields. In food and drug safety, this technology has the potential to address key adulteration challenges: further quantifying specific species in food supplements or processed pharmaceuticals to prevent mislabeling and safeguard consumer health [18, 24]. In forensics, it has the potential to expand upon the successful application of pyrosequencing-based SpeID technology in identifying animal hair tissue [12], enabling the quantification of plant residues (e.g., herbal extracts in toxicological analyses or plant fragments at crime scenes) to support investigations. In biodiversity monitoring, its advantage in distinguishing low-taxonomic-level closely related species—overcoming limitations of HRM [31] and ddPCR [2, 34] and has the potential to accurately quantify target species in ecological samples. Additionally, for regulatory compliance, it has the potential to provide a standardized, accurate detection method aligned with international pharmacopeia requirements (e.g., distinguishing herbal species in Chinese, European, and US pharmacopeias), helping regulatory agencies enforce quality standards for herbal medicines and related products.

In previous studies (e.g., those on *P. ternata* or Fritillaria thunbergii) and the present study, we have identified limitations of the Herb-Q assay: specifically, it exhibits relatively high limits of detection and quantification (LOD/LOQ) at 2%, and this limitation mainly stems from two factors—first, the Herb-Q assay involves a multi-step workflow covering the process from DNA extraction to the generation of final quantitative results, and second, it relies on a pyrosequencing system (instrument platform) that is currently unable to output results in decimal form with a decimal point [12, 16], it should be noted that although this error spans the entire process from DNA extraction to final quantitative results and its level is considered acceptable given the complexity of the analysis, and the average deviation in this study is also significantly lower than the reference deviation value of 25% [9] and the 7.3% deviation reported in other molecular method studies [36], addressing this limitation has still been listed as a top priority for future development to obtain more accurate quantitative results. Furthermore, developing portable systems through platform miniaturization and simplified data processing (e.g., cloud-based workflows) is another key priority, as this advancement is crucial for establishing methods that enable convenient on-site testing.

## Conclusion

This study demonstrated the effectiveness of the Herb-Q assay for precise quantitative analysis of edible-medicinal licorice species and introduced an SLS algorithm system tailored for screening detection loci in the Herb-Q assay. The process demonstrated a 100% accuracy rate in the species identification of medicinal licorice within the scope of this study, based on the tested samples. The LOD and LOQ of this method were 2%, and it showed excellent linearity and accuracy. The average bias of the detection of mixed powders and Chinese patent medicines were low, with average discrepancies of 8.25% for GG and 8.01% for GU in the mixed powder. When only GG is present in Liuyi San, the bias was 6.43%. With both GG and GU in Liuyi San, the discrepancies for GG and GU were 5.61% and 3.48%, respectively. The Herb-Q assay holds great promise for accurately quantifying individual species in agricultural products, foods, and environmental samples, etc. The establishment of the SLS algorithm system is of great significance to the rapid implementation and promotion of this assay, paving the way for its widespread use in the future.

## Supplementary Information

Additional file 1.

Additional file 2.

Additional file 3.

## Data Availability

The data underlying this article are available in the article and in its online supplementary material.

## Abbreviations

SLS: Specific loci screening
GG: *Glycyrrhiza glabra*
GU: G. *uralensis*
GI: G. *inflata*
Herb-Q: Herb molecular quantification
HRM: High-resolution melting analysis
SSRs: Simple sequence repeat markers
cp: Chloroplast
LOD: Limit of detection
LOQ: Limit of quantification
SNP: Single nucleotide polymorphism
