# Supplementary material for: A novel algorithm- specific loci screening accelerates the establishment of molecular quantification of Glycyrrhiza glabra, G. uralensis, and G. inflata
Source: Chin Med. 2025 Nov 24;20:200. doi: 10.1186/s13020-025-01263-2 (PMC12642167; doi:10.1186/s13020-025-01263-2)
Supplement: Supplementary file 1 — Additional file 1. [file 13020_2025_1263_MOESM1_ESM.zip › Supplementary code files/Details of the SLS algorithm.docx]

**Details of the SLS algorithm**

The SLS algorithm, as implemented in the provided codebase (Algorithms 1–4), involves a series of computational steps with specific parameters and thresholds, complemented by a structured validation process. Below are the detailed descriptions:

**1. Computational Steps**

The algorithm is designed for sequence comparison and feature extraction from genetic data (e.g., FASTA files) and involves four core stages:

**Step 1: Data Parsing (Algorithm 1)**

Objective: Convert raw FASTA files into structured dictionaries for downstream processing.

Parameters: Input file path (./sequence.fasta) and output directory (./).

Process: Parses FASTA headers to generate unique identifiers (IDs) for each sequence (e.g., combining genus, species, and accession information) and concatenates sequence fragments into complete strings.

**Step 2: Sequence Comparison (Algorithms 2 and 3)**

Objective: Identify discriminative positions between target and reference sequences.

Key Parameters:

is_gang: A boolean flag (True/False) controlling whether to include sequences containing the gap character ('-').

query_flag and compare_flag: Lists of sequence identifiers (e.g., ['Glycyrrhiza_glabra'], ['Glycyrrhiza_']) to define target (query) and reference (comparison) groups.

max_len: Maximum sequence length (e.g., 155686 in Algorithm 2, 137214 in Algorithm 4), determining the range of positions to analyze.

Thresholds for Filtering:

In method and method_2 (Algorithms 2 and 3):

All query sequences at a position must be identical (len(set(querys)) == 1).

No overlap between query and comparison sequences at the position (len(set(compares) & set(querys)) == 0).

Exclusion of positions with gaps if is_gang=False ('-' not in querys and '-' not in compares).

**Step 3: Feature Validation (Algorithm 3)**

Objective: Refine candidate positions using quality control thresholds.

Consecutive: Positions are rejected if the maximum length of consecutive identical characters in a 27-bp window (±13 bp around the position) exceeds 4 (max(sub_max_dup_len) ≥ 4).

Neighbor consistency filtering: Positions are rejected if the first two flanking positions (±1, ±2 bp) show non-uniformity (max(len_[:2]) > 1).

Cumulative difference filtering: Positions are rejected if more than 5 out of the next 13 flanking positions (±3 to ±15 bp) show non-uniformity (sum(clac_sum) > 5).

**Step 4: Annotation and Classification (Algorithm 4)**

Objective: Map positions to genetic features (e.g., genes) and classify results.

Parameters: GenBank (.gb) files for gene annotation, with indices mapped to gene names via index_dict.

Process:

Converts raw positions to gap-adjusted positions (excluding '-').

Classifies positions into need (informative, no gaps and non-uniform) and no_need (uninformative, with gaps or uniformity) via state_2_process_tst.

**2. Validation Process**

The algorithm’s validation relies on iterative filtering and result segmentation:

Intermediate Outputs: Generates stage-specific files (e.g., all_result_stage_1.xlsx, all_result_stage_2.xlsx in Algorithm 4) to track position annotations and uniformity.

Pass/Fail Classification: Outputs result_yes_pass.xlsx (positions meeting all thresholds) and result_no_pass.xlsx (rejected positions with failure reasons, e.g., "the first step" for consecutive repeats) in Algorithm 3.

Consistency Checks: Ensures reproducibility by fixing parameters (e.g., window sizes, thresholds) across runs and validating against reference sequences with known genetic features.

These steps collectively ensure that the SLS algorithm robustly identifies biologically meaningful genetic variations while minimizing noise from gaps or repetitive regions.
