# Supplementary material for: A novel algorithm- specific loci screening accelerates the establishment of molecular quantification of Glycyrrhiza glabra, G. uralensis, and G. inflata
Source: Chin Med. 2025 Nov 24;20:200. doi: 10.1186/s13020-025-01263-2 (PMC12642167; doi:10.1186/s13020-025-01263-2)
Supplement: Supplementary file 2 — Additional file 2. [file 13020_2025_1263_MOESM2_ESM.docx]

**Quantification of original species of medicinal licorice using Herb-Q assay**

Yifei Pei^a^, Ziyi Liu^a^, Wenjun Jiang^a^, Mingyu Zhang^a^, Haitao Liu^b^, Xue Feng^a,^ * and Xiwen Li^a,b^ *

^a^ *State Key Laboratory for Quality Ensurance and Sustainable Use of Dao-Di Herbs, Institute of Chinese Materia Medica, China Academy of Chinese Medical Sciences, Beijing 100700, China*

^b^ *Institute of Medicinal Plant Development, Chinese Academy of Medical Sciences, Peking Union Medical College, Beijing 100193, China*

**Authors**

**Yifei Pei**, Email: yfpei@icmm.ac.cn. ORCID: 0000-0002-3638-9017

**Ziyi Liu**, Email: liuziyi20210121@163.com. ORCID: 0009-0003-2566-2203

**Wenjun Jiang**, Email: wenjunjiang0927@gmail.com. ORCID: 0000-0001-6490-9449

**Mingyu Zhang**, Email: zmy421317@163.com. ORCID: 0009-0007-9361-4392

**Haitao Liu**, Email: htliu@implad.ac.cn. ORCID: 0009-0002-4342-3938

**Xue Feng**, Email: xfeng0413@icmm.ac.cn. ORCID: 0000-0003-0334-1225

**Xiwen Li**, Email: xwli@icmm.ac.cn. ORCID: 0000-0002-5092-854X

***** **Corresponding author**

**Xiwen Li**, Institute of Chinese Materia Medica, China Academy of Chinese Medical Sciences, Beijing 100700, China.

Tel +86 010 8408 4107; fax +86 010 8408 4107.

E-mail: xwli@icmm.ac.cn (X.L.)

**Xue Feng**, Institute of Chinese Materia Medica, China Academy of Chinese Medical Sciences, Beijing 100700, China.

Tel +86 010 8408 4107; fax +86 010 8408 4107.

E-mail: xfeng0413@icmm.ac.cn (X.F.).

**Short legends for Supporting Information**

**Figure S1** Verification of the specific single nucleotide polymorphism loci in GG, GU, GI and the mixture at the 12 loci. GG, *G. glabra*. GU, *G. uralensis*. GI, *G. inflata*. The mixture is a mixture of 3 kinds of medicinal licorices in a ratio of 1:1:1.

**
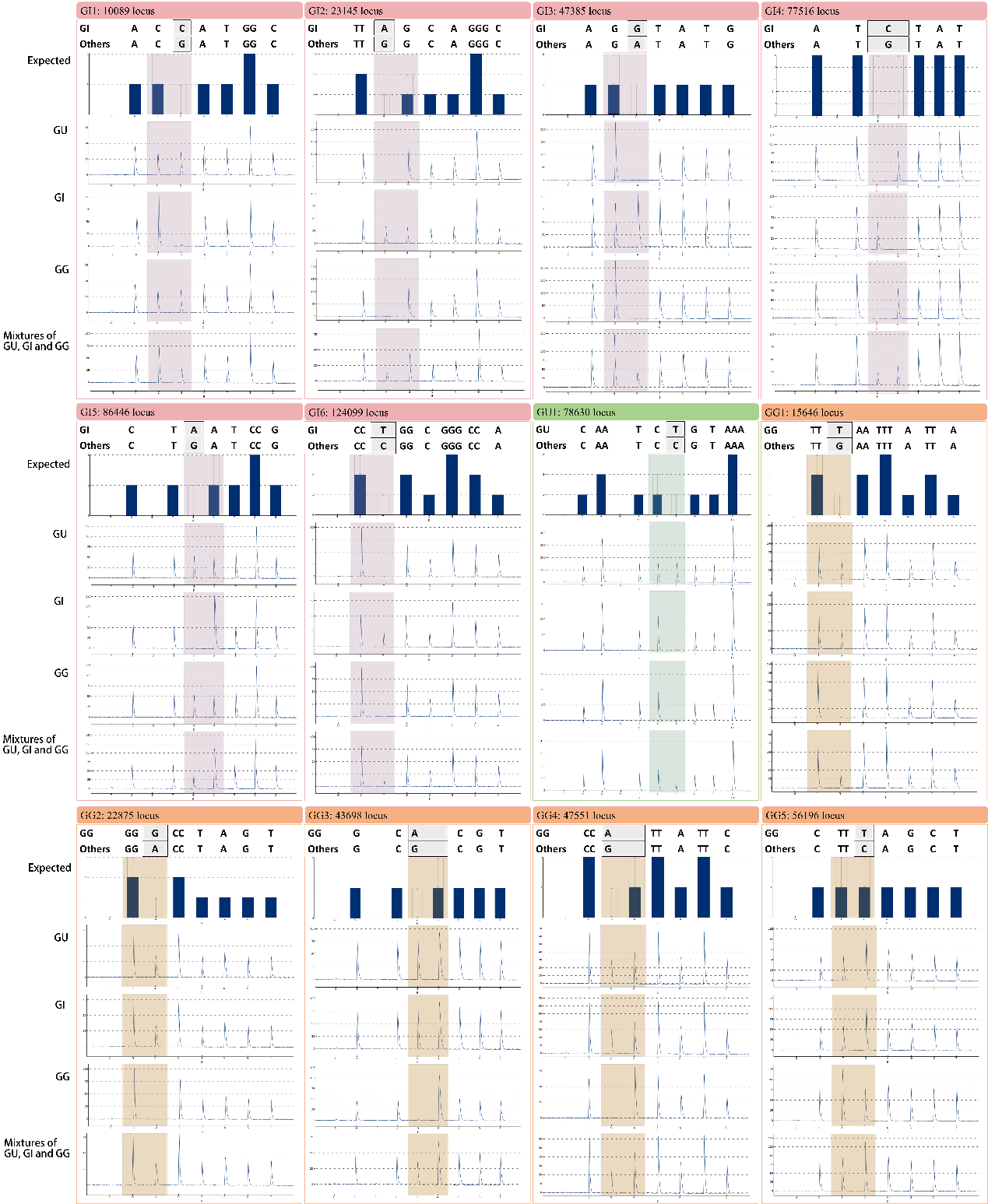
**

Figure S1
